# Report on Test Fillings Made by Several Operators by Several Methods at Wisconsin State Meeting

**Published:** 1910-05-15

**Authors:** 


### REPORT ON TEST FILLINGS MADE BY SEVERAL OPERATORS BY SEVERAL METHODS AT WISCONSIN STATE MEETING.

There were eight men who entered in this Test Clinic No. 1. The first on the list was Dr. Doyle who used annealed Watts' gold and Rowans' pellets. Dr. Doyle used hand pressure; the time consumed was 31¼ minutes. Tested by air for leakage it showed no leakage whatever under sixty pounds pressure. Tested for strain it yielded at thirty-three pounds.

The second on the list is Dr. Harold E. Holbrook. He used Pack's cylinders, hand pressure and automatic mallet; time consumed 37¼ minutes; showed leakage at forty-three pounds pressure; yielded to strain at thirty-two pounds.

The third was Dr. M. L. Christensen. He used Pack's cylinders; automatic mallet; time consumed 36½ minutes; showed a very slight leakage at nine pounds pressure, at another point it showed leakage at thirty-six pounds. It did not pull out under the strain, that is, the filling did not part, but the whole tooth broke at sixty-four pounds.

Fourth on the list was Dr. Wm. Hopkinson. He used Hood's gold and Hood's pellets; I believe the first was unannealed and the second annealed. Method, hand mallet; time consumed 21¾ minutes; showed slight leakage at sixty pounds air pressure. Yielded to strain at forty-eight pounds.

Dr. E. A. Geilfuss was the fifth. He used Rowan's cylinders and Ney's foil; method, Bonwill mechanical mallet; time consumed twenty-eight minutes; showed slight leakage at thirty-five pounds air pressure, and the peculiarity of this, which I wish to mention, is that this leakage was not at the margin, but was through the gold. This leakage did not increase with the increase in pressure and was only slight as it did not effervesce as the pressure increased. It yielded to strain at thirty-four pounds.

Dr. W. J. Claasen used Watt's gold, hand pressure and hand mallet; time consumed thirty-seven minutes; showed slight leakage at fifty-three pounds pressure. Yielded to strain at seventy-six pounds.

Dr. L. J. Stephan used Pack's cylinders and Ney's foil; method, Bonwill mechanical mallet; time consumed 36½ minutes; showed very slight leakage at nineteen pounds and leakage did not increase with any pressure. Yielded to strain at thirty-eight pounds.

Dr. P. B. Wright used Watt's gold; hand and automatic mallet; time consumed, thirty-five minutes, showed slight leakage at twenty-five pounds pressure, increased very slightly at forty pounds and yielded to strain at twenty-four pounds.

### REPORT OF THE JUDGES ON CLINIC NO. 2.

In this clinic fillings were made in previously prepared large cavities in the lingual aspect of the crowns of fresh bovine incisors so prepared as to involve the incisal edge. The teeth were from the same animal and had been kept moist. As nearly as possible uniformity of cavities was secured.

FILLING NO. 1.

Operator—Dr. R. E. Maercklein, Milwaukee, Wis.

Method—Electric Mallet.

Kind of gold—Ney's cohesive No. 3 folded.

Time of packing—28 minutes 15 seconds.

Condition of filling before finishing—Considerably more than full.

Time required for finishing—14 minutes, 15 seconds.

Condition of contour and margins—Overfull on lingual surface and along all lingual margins. Labial good except slight defect at one margin. Incisal edge of gold slightly convex.

Leakage—At 14 pounds leaked in 2 places, at gingival, and at 24 pounds leaked profusely at incisal.

Specific gravity—At 60 degrees F. 14.90.

FILLING NO. 2.

Operator—Dr. L. A. Meyer, Oconomowoc, Wis.

Method—Mechanical Mallet.

Kind of gold—Pack's cylinders.

Time of packing—36 minutes, 30 seconds.

Condition of filling before finishing—Slightly over full.

Time required for finishing—15 minutes.

Condition of contour and margins—Contour good. Slightly overfull on lingual margin. One slight concavity on lingual, labial good.

Leakage—At 24 pounds leaked at one place on labial surface, and did not change materially up to 60 pounds.

Specific gravity—At 60 degrees F. 17.73.

FILLING NO. 3.

Operator—Dr. A. C. Searl, Owatonna, Minn.

Method—Mallet in hand of assistant.

Kind of gold—Cohesive pellets.

Time of packing—42 minutes, 50 seconds.

Condition of filling before finishing—Slightly under full.

Time required for finishing—11 minutes, 15 seconds.

Condition of contour and margins—Slightly concave on lingual, one slight defect on labial—not quite full—small pit.

Leakage—At 4 pounds leaked in one place, at 7 pounds 2 places at incisal, at 24 pounds 3 places at incisal.

Specific gravity—At 60 degrees F. 15.55.

The specific gravity was made by J. S. Janssen, Ph. D., and reported back to the judges. As a matter of additional interest the specific gravity was made of a filling inserted by Dr. B. G. Maercklein and worn in a left upper central incisor for twelve years when the tooth was broken by a blow on the filling. This was inserted by the electric mallet and showed a specific gravity of 18.48.

In the clinic in question the operators worked under the disadvantage of being away from their usual environment and of working outside of the mouth, but with all of this, considering the conditions, they performed most creditable operations, and the Judges wish to extend to them their thanks for their loyal aid in making the Clinic a success. They are all operators of a high order of skill and the Society was fortunate in securing their services.

Signed:

C. N. Johnson,
Arthur D. Black,
B. G. Maercklein.

### DISCUSSION OF TESTS.

Dr. A. D. Black.—There are several things in connection with the placing of a gold filling, that a man should bear in mind. Specific gravity of a filling is one thing; and the adaptation of the gold to the cavity walls is another thing. We may mallet gold ever so hard and make a very hard gold filling, and yet an absolutely worthless one. We may mallet a filling more lightly and yet make a splendid filling if the adaptation is right. We may have a filling with a very high specific gravity that is not a good filling. The question of placing a good gold filling does not depend upon any one thing, but upon the application of the proper knowledge of all things necessary in each particular case. In considering the condensation of gold, there are several items that we must take into consideration. The three most important are: the force of the blow that we deliver; the size of the plugger point we use, and the one thing that too many of us forget, the resistance that is offered to the blow by the tooth as it stands in its socket. We must mallet harder or must have smaller plugger points in packing gold into the cavity in the tooth of a child, because the peridental membrane supporting such a tooth allows more movement of the tooth than is the case in the older person.

A tooth is supported by fibres and if we strike a blow with any kind of a mechanical mallet, we strike the tooth as it stand suspended in its socket. If we can render the fibres of the peridental membrane tense before the blow is struck, we will get a better result, In other words, if the operator will, before each blow is struck, make pressure on the tooth with his plugger point, the tooth will offer more resistance to the blow and he will get greater condensation of the gold from a given blow. This I consider a decided advantage to be gained by the use of the hand mallet by an assistant.

I want to remind you again that the area of the end of the plugger point is a very important thing to consider in condensing gold. If you double the diameter of the plugger point; if you change from a plugger one millimeter in diameter to a plugger two millimeters in diameter, you must strike four times as hard a blow to get the same condensation of gold. Therefore, by adjusting your plugger points, larger or smaller, to the particular case in hand, according to the resistance offered, you can by striking more and lighter blows on the smaller point get the same results that you would with less number and heavier blows on a larger point.

One of the interesting features of the experiments reported here today is that all but two of them showed imperfect adaptation of gold. I want to lay particular emphasis on the fact that the dental profession as a whole does not pay enough attention to the proper direction of the force used to adapt the gold to the wall of the cavity. That must apply to every part of the wall in all portions of the cavity, because the leakage in any one place is just as bad as if it leaked everywhere.

I want to mention five things that we should think about in placing gold fillings. First: We ought to think about how we will lay on each piece of gold, that is, how we will build up our fillings in order to keep our gold in the most solid mass. We must keep it in as compact a mass as possible, and not spread it out in a thin layer over any wall or margin. Second: We should study the direction of the force as we apply it, in order to get a proper adaptation of that gold. Third: In order to get the greatest density, we should have some definite manner of stepping the plugger over the piece of gold, so that we do it in a systematic way. Fourth: We should study the proposition of wedging the gold against the dentine so as to take advantage of the tremendous elasticity of the dentine. Fifth: We should study carefully the size of the plugger points so as to get the best possible condensation with the least pain to the patient. We should also study the force of the blow that should be delivered on the plugger points in order to get the proper condensation.

The experiments we have witnessed here today and the papers that have been read, should set each one of us thinking for himself to the end that we will appreciate the fact that if we are going to make fillings that will last, we must study all of these things in connection with every filling that we make.

Dr. C. N. Johnson.—The thing that impresses me more than anything else is this: that we cannot get away from the personal equation. I would be willing as a patient, and from the point of view of the patient, to take the chair of a man who is conscientious and who has become accustomed to one method of operating and accept his experience. These things can be reduced through study to a mechanical exactness and the tests that have been made today have been along that line. I wish that Dr. Southwell were here in order that I might express my thanks for this work. The question of principle comes in in all of our work, and I know that if we have studied the fundamental basis of the proper condensation and adaptation of gold, we must rely upon that particular method which in our individual hands will give the best results. In other words, we must put intelligence into our operations. When we study carefully the results of our operations from our records of operations made in the teeth in the mouth we have a basis for guidance which I believe is safer than all others. That brings me to the question that I believe more important than the tests and that is, that records must be carefully kept. A man should be able to turn to any operation and know precisely the result of that operation, know the circumstances under which it was performed and be able to base his future practice on the years of experience he has put in at the chair. It is the work that we do at the chair that is the most significant.

I want to express my appreciation of the operators in clinic No. 2, and I say to you in all frankness, that if I had a tooth to fill, I would shut my eyes, put myself in the chair and let either one of them fill it.

One of the requisites of a good gold filling is to seal the cavity against leakage. You may have a filling that is hard, but if it leaks it is not a good filling and it will not do service very long. You may have a filling that is of high specific gravity; if it leaks you will not have a good filling. The thing to remember first of all is to seal the cavity. There are a great many things that must be considered; we must have sufficient specific gravity to make the filling stand up under the stress exerted upon it during mastication. I would not expect a filling to stand up under severe usage with a specific gravity lower than 14. The form of the filling is of great importance when the comfort of the patient is considered. Our work should be based on the comfort of the patient in mastication.

I want to say another thing, if I may, and that is that in your present reorganization plan you have a problem before you. The interest has grown immensely now and it remains for you to maintain that interest. The danger is that your interest will relax. Keep it up to the highest point. You have a great opportunity to make a record here. We in Illinois will run a race with you, and it will be a lively one and we will make you go some. Develop your component societies; work with that idea in your minds and remember always that they are the most important factor in your success.

DR. MAYER.—I rise to defend the mechanical mallet with Dr. Southwell. I wish to say that I have been operating with the mechanical mallet for seventeen years and with entire satisfaction to myself and I consider that I am giving to my patients value received. It takes time and perseverance to use the mechanical mallet and get out of it what is in the instrument. I do not contend that the mechanical mallet is the only mallet that can be used to put in a perfect filling. As far as the plugger is concerned, I have used foot plugger S. S. White No. 47 ever since I used the mallet. I have had it manufactured with some modifications to suit my various needs. It is a very small one. It has very fine serrations. Dr. Southwell uses one of his own pattern which is similar. I feel that I get very good results and he also does that. It is a matter of individuality in operating. We all have our own way.

DR. WENDEL.—In the preparation of these teeth a drill was used that would only cut a certain depth and as Dr. Johnson has told you, the pulp chamber was encroached upon. These cavities were not prepared along the same lines that an operator would prepare them in the mouth. The main object was to make them of a uniform depth.

I have practically used the electric mallet for thirty years and those who know me well know that I have always been an upholder of that instrument. I do not say that it is the only instrument that can pack gold or produce a good filling. I agree with Dr. Johnson in what he says that if a man can produce the best results with a certain instrument, he had better cling to the instrument that he is accustomed to than deviate from it and take up another instrument that he cannot handle with the same ease and to the best advantage. I am not going to discuss scientific lines nor mechanical lines. You have heard it from the different speakers here. There is one thing I am interested in, however. I heard Dr. Wedelstaedt's remark regarding the efficiency of a lady assistant, and I want to know who it is, his lady assistant or himself who is responsible for the filling. A hypnotic influence must necessarily take place between the lady assistant and himself if the blow is to be struck spontaneously, the same as a mechanical instrument. The electric mallet is rapid and quick in stroke and, therefore, great care should be taken and I think that it can produce the best results with 32, 36 or 48 gold in ribbon form.

Is the lady assistant striking the sort of blow that the operator desires at all times? Sometimes your patient will indicate it to the operator or to the lady who is striking the blow.

Dr. Maercklein has presented to you a filling of 18. specific gravity, showing absolutely that gold with the electric mallet can be perfectly packed by a good operator. The trouble usually lies with the operator. I think that is true of the hand mallet also, as well as with the mechanical mallet. I have seen the most beautiful fillings made with a hand mallet.

Dr. Maercklein.—I desire to make only a few remarks but I would like to repudiate the statement of Dr. Wedelstaedt that the electric mallet does not weld the gold. In the tooth I showed you the filling remained intact when it was broken off until lately when the tooth split in two pieces and the filling came out. A number of the members of the Society saw it some years ago at Madison. I maintain that gold which is not welded could not have stood the strain. You can weld gold just as perfectly with the electric mallet when it is properly adjusted and handled, as you can with any other instrument. I do not say that you can do it better, but you can do it as well. This filling was in the mouth for more than twelve years.

Dr. Wedelstaedt.—Last year George Ade took the most successful of his plays over to London. They fell perfectly flat and he and his supporters lost all that they had made in this country. When they returned to New York they were one evening sitting in a cafe with a number of other men complaining very bitterly of their loss. Ade made some disparaging remarks regarding an Englishman's ability to see a joke. There was an Englishman present at the table. He said, "You can always tell an Englishman wherever you see him." "Yes," said Ade, "You can tell him, but that is all the good it does you."

Now, Dr. Maercklein is one of the best fellows I know; he is a good deal like myself. He has had so much experience that he bases his ideas and his opinions on it. I do the same thing. Nothing which I could say would in the least influence Dr. Maercklein to change his ideas. If he would take me to his office, make a demonstration and let me see him operate, I might again give the electric mallet a trial, but it would not perhaps be as successful an instrument in my hands as it would be in the hands of one of unquestioned ability in the use of the instrument, like Dr. Maercklein. As one gentleman has just said an acquaintance with the instrument is a very important factor in this matter. I know how difficult it is to make operations in cavities in the human teeth. I know it very well. I know my own shortcomings better than anybody else, but nothing has so far been said that will cause me to change my ideas.

I want to tell you a little story. It is an actual occurrence, by the way. Dr. Searl and I were holding a school in an adjoining city. There were a number of men operating under our supervision. As I walked down the room, I stumbled over something and turned round to find that the cause was a series of dry cells, forming a battery, which were on the floor. I looked at the man at the chair and asked him what he was doing. He said, "I am packing gold and using the only instrument that a sane man should use." I looked at the man and at the large foot plugger he was using. I called my assistant, took a 5-10 millimeter plugger and asked if I might show him something. I took an area of two millimeters in the cavity he was filling and sunk a pit right into the gold. He discontinued using the electric mallet and went to using a hand mallet. I am not saying that I could do this to a filling that Dr. Maercklein is condensing, but on several occasions since then, I have done the same thing.

There is one statement which was made, in the first essay read, that needs some further discussion. The essayist says that one beauty in using the electric mallet is that the plugger may be held some distance away from the gold while it is being used for condensing the different masses of gold which are placed in the cavity. I am unable to bring myself into that condition where this statement can be considered in the light which the essayist desires. We wish blows on the plugger for the purpose of welding, toughening and making the surface of the gold hard, therefore, the plugger point should be held firmly against the mass of gold that is being condensed. I wish to illustrate this: we drive nails in boards. Do we in doing this hold the nail away from the board prior to striking it with the hammer or do we hold the nail firmly against the board? Well, what do we do it for? You all know. The self-same thing applies where we are condensing gold. If the best results are desired the plugger should be held firmly against the gold and not a little distance from it.

I believe that from 10 to 20 per cent more gold may be condensed in a cavity by holding the plugger firmly against the gold, while condensing it, than can be condensed into the same cavity where the plugger is held some distance away from the gold during the condensation. If, as the gentleman says, that where teeth are sore and tender, the peridental membrane being inflammed, if it were necessary to operate under such conditions, I should adjust a separator and thus hold the tooth firmly in position. I should not care to jeopardize the stability of the operation by not thoroughly condensing the gold.

The question has been asked, "Who is responsible for the filling, the assistant or the operator?" I do not question for a moment that the operator is always responsible. He is the one the patient looks to and not to the assistant. I have merely to imply my wish and my assistant knows what is desired. So closely will your assistant learn your ways and learn to mallet that she will do this instinctively and effectively for she will know from your slightest move what is desired. A nod of the head will bring a blow that is strong; another movement of the head and there is a diminution of the blow. There has been something said about fillings remaining in the teeth. I am never interested in operations which are doing good service. What I am interested in, and what I study all the time and think about morning, noon, and night, are the failures which come to me. The length of time a filling has been in a tooth and has been doing good service, I am not interested in in the least.—*Dental Review.*

